# The Role of Muscle Strength, Physical Activity, Perceived Stress, and Sleep Quality in Patients with Hypertension

**DOI:** 10.3390/jfmk11010112

**Published:** 2026-03-06

**Authors:** Veronica Potosi-Moya, Ronnie Paredes-Gómez, Shulianna Burgos-Vera, Samantha Báez-Narváez

**Affiliations:** 1 Facultad Ciencias de la Salud, Universidad Técnica del Norte (UTN), Ibarra 100105, Ecuador; vjpotosi@utn.edu.ec (V.P.-M.); bsburgosv@utn.edu.ec (S.B.-V.); snbaezn@utn.edu.ec (S.B.-N.); 2 Red de Investigación en Fisioterapia (RIF), Ibarra 100105, Ecuador

**Keywords:** blood pressure, muscle strength, quadricep strength, hamstring strength, physical activity, perceived stress, sleep quality

## Abstract

**Background:** Hypertension is a multifactorial condition influenced by physiological, behavioral, and psychosocial factors. Muscle strength, physical activity, sleep quality, and perceived stress may contribute to blood pressure variability, although their relative influence remains unclear. This study examined associations between systolic blood pressure (SBP) and demographic, anthropometric, neuromuscular, behavioral, and psychosocial variables in adults with primary hypertension, with secondary analyses for diastolic blood pressure (DBP) and sex differences. **Methods:** A cross-sectional study was conducted in 391 adults with hypertension (165 men, 226 women). Predictors included age, body mass index, lower-limb muscle strength, physical activity (GPAQ), sleep quality (PSQI), and perceived stress. Associations were analyzed using correlation analyses and sex-stratified multivariable regression models. **Results:** In men, SBP correlated positively with age and negatively with lower-limb strength. In women, SBP showed associations with physical activity and perceived stress. Regression analyses indicated that sleep quality and perceived stress were independently associated with SBP in women (adjusted R^2^ = 0.13), whereas hamstring strength was associated with DBP in men with low explanatory capacity (R^2^ = 0.05). Moderate-to-high collinearity was observed among strength variables. **Conclusions:** Blood pressure variability was associated with neuromuscular and psychosocial factors with sex-specific patterns; however, the modest explained variance suggests these factors act as complementary rather than primary determinants. Longitudinal studies are needed to clarify causal relationships.

## 1. Introduction

Hypertension (HTN) is one of the leading modifiable risk factors for cardiovascular morbidity and mortality worldwide, affecting more than one billion individuals and contributing substantially to the global burden of disease. Elevated blood pressure is strongly associated with adverse cardiovascular outcomes, including stroke, myocardial infarction, and chronic kidney disease. Despite advances in pharmacological treatment, blood pressure control remains suboptimal in many populations, highlighting the importance of identifying additional modifiable factors that may contribute to blood pressure regulation and cardiovascular risk reduction [1].

Blood pressure regulation is influenced by multiple physiological and behavioral factors that interact through complex mechanisms involving vascular function, autonomic nervous system activity, and metabolic regulation [2]. Muscular strength and physical activity have been associated with improved endothelial function, enhanced insulin sensitivity, and reduced systemic inflammation, which may contribute to better blood pressure control [3]. Furthermore, psychosocial factors such as perceived stress and sleep quality can modulate sympathetic nervous system activation and hormonal responses, including cortisol secretion, thereby influencing cardiovascular regulation and blood pressure levels [4,5]; consequently, blood pressure should be considered within a multidimensional framework integrating physical, behavioral, and psychosocial determinants.

Body mass index (BMI) is a well-established risk factor for hypertension and cardiovascular disease, influencing vascular structure, metabolic regulation, and insulin sensitivity from early adulthood [6]. Excess adiposity has been associated with increased sympathetic activity, endothelial dysfunction, and vascular remodeling, which may contribute to elevated blood pressure levels. However, lifestyle modifications, including weight reduction and increased physical activity, have demonstrated beneficial effects on blood pressure regulation and cardiovascular risk reduction [7,8]. Given the complex interaction between metabolic, behavioral, and physiological factors, hypertension should be understood as a multifactorial condition influenced by both body composition and modifiable lifestyle determinants [9]. These metabolic alterations are also closely related to components of the metabolic syndrome, which may further contribute to cardiovascular risk and blood pressure dysregulation.

Physical inactivity has progressively increased worldwide over recent decades, resulting in significant public health implications. Currently, approximately one in four adults is classified as physically inactive, with women being less active than men, and populations from low- and middle-income countries being disproportionately affected, contributing to elevated cardiovascular and metabolic risk [10]. Sedentary behavior and prolonged sitting time have been associated with impaired vascular function, reduced metabolic efficiency, and increased blood pressure levels due to low energy expenditure and autonomic imbalance [11]. Conversely, regular physical activity has demonstrated beneficial effects on cardiovascular regulation, including improvements in endothelial function, autonomic balance, and systemic inflammation, supporting its role as a modifiable factor in blood pressure control. The magnitude of these cardiovascular benefits appears to depend on the type, intensity, and dose–response relationship of physical activity, highlighting the importance of considering qualitative aspects of exercise behavior [12,13].

Adequate sleep quality is also of great importance for individuals with hypertension, as physiological rest during sleep is associated with nocturnal blood pressure reduction and cardiovascular recovery. Conversely, sleeping less than six hours or experiencing fragmented sleep has been associated with increased daytime fatigue, elevated cortisol levels, higher blood pressure values, and increased heart rate [14], as well as impaired endothelial function, which is directly related to cardiovascular health [15,16]. These alterations suggest that sleep disturbances may contribute to dysregulation of autonomic and hormonal mechanisms involved in blood pressure control.

Psychological stress plays a significant role in blood pressure regulation through multiple physiological pathways that adversely affect cardiovascular function. Acute stress activates the “fight-or-flight” response mediated by sympathetic nervous system stimulation and hypothalamic–pituitary–adrenal (HPA) axis activation, leading to transient blood pressure elevations through vasoconstriction and increased secretion of stress-related hormones. However, persistent exposure to stress and prolonged cortisol elevation generates cumulative physiological burden that becomes maladaptive and has been associated with hypertension development and progression [17].

In individuals with hypertension, muscular strength may be negatively affected, particularly in antigravity muscles and those with high blood flow, such as the quadriceps, gluteus maximus, and hamstrings [18]. Muscular strength is increasingly recognized as a cardiometabolic risk marker and may serve as a predictor of morbidity and mortality in the general population. Greater upper- and lower-limb strength has been associated with a lower risk of cardiovascular disease, whereas reduced strength is linked to increased cardiovascular risk [19,20]. Furthermore, evidence supports the use of strength-training protocols involving sustained contraction of major muscle groups, which have been associated with improvements in blood pressure regulation, muscle mass, sleep quality, functional capacity, and overall quality of life in the short and medium term [21,22,23,24].

However, there is still limited evidence integrating muscular, behavioral, and psychosocial variables simultaneously, such as lower-limb strength, physical activity level, perceived stress, and sleep quality, in relation to blood pressure in hypertensive populations. Most previous studies have evaluated these factors independently [25], and the relative contribution of each component within a multidimensional framework remains unclear. Understanding the combined contribution of these factors may improve risk stratification and therapeutic strategies in hypertensive populations. Therefore, the primary objective of this study was to examine the association between systolic blood pressure (SBP) and demographic, anthropometric, neuromuscular, behavioral, and psychosocial variables in adults with recently diagnosed hypertension. SBP was de-fined as the primary outcome variable. Secondary objectives included the evaluation of associations with diastolic blood pressure (DBP) and the exploration of potential sex-specific differences through stratified analyses. We hypothesized that muscle strength, physical activity, sleep quality, and perceived stress would be independently associated with SBP after adjustment for age and body mass index.

## 2. Materials and Methods

### 2.1. Study Design

This study employed an observational analytical cross-sectional design conducted in adults diagnosed with primary hypertension. Participants were recruited from public healthcare institutions in the province of Imbabura, Ecuador, during 2024 and examined the association between systolic blood pressure (SBP) and diastolic blood pressure (DBP) and functional, behavioral, and psychosocial variables, including lower-limb muscle strength, physical activity level, perceived stress, sleep quality, age, sex, and body mass index.

The inclusion criteria were adults aged 40–78 years with a confirmed medical diagnosis of primary hypertension who were receiving care at public healthcare institutions and were able to safely perform the physical assessments.

Exclusion criteria included secondary hypertension, hypertension-mediated organ damage, diabetes mellitus, uncontrolled hypertension at rest, severe musculoskeletal or neurological disorders limiting functional performance, knee pain restricting physical activity, pregnancy or breastfeeding, and any contraindication to physical exertion according to clinical evaluation.

### 2.2. Study Population

A total of 391 adult patients with a medical diagnosis of grade 1 hypertension were enrolled, defined as systolic blood pressure (SBP) between 140–159 mmHg and/or diastolic blood pressure (DBP) between 90–99 mmHg according to current international clinical criteria. Participants were consecutively recruited during outpatient visits from the general medicine, internal medicine, and cardiology departments of public healthcare centers authorized by the Ministry of Public Health throughout the study period. All eligible patients received detailed information regarding the study procedures and provided written informed consent prior to enrollment.

Eligibility criteria were defined previously (Section 2.1).

The consecutive inclusion of patients from multiple healthcare centers allowed the incorporation of a clinically heterogeneous sample representative of individuals receiving care within the public health system. However, probabilistic sampling was not performed, as recruitment was limited to patients attending healthcare services during the study period. Therefore, the findings should be interpreted within the clinical context of the studied population.

### 2.3. Measurements and Instruments

Body weight was measured using an Omron M6 Comfort (Omron Healthcare Co., Ltd., Kyoto, Japan) digital scale, and height was measured using a portable stadiometer with participants barefoot and wearing light clothing. BMI was calculated as weight divided by height squared (kg/m^2^). BMI was analyzed as a continuous variable and additionally classified according to World Health Organization criteria: underweight (<18.5 kg/m^2^), normal weight (18.5–24.9 kg/m^2^), overweight (25.0–29.9 kg/m^2^), and obesity (≥30 kg/m^2^) [26].

Blood Pressure (SBP and DBP): Blood pressure was measured using a calibrated Omron M6 Comfort digital sphygmomanometer (measurement range 0–300 mmHg, Omron Healthcare, Shangai, China) with a standard cuff. Measurements were performed following the standardized protocol of the Pan American Health Organization (PAHO) [19,27], Participants were seated in a chair with back support, feet flat on the floor, and rested for 5 min prior to the first reading. The cuff was placed on the upper arm with the forearm supported in supination, the device was activated, and the measurement was recorded. Two readings were taken, separated by a 5-min interval, and the mean value was used for analysis [28]. This device is included in the HEARTS in the Americas validated list of automatic BP measuring devices, endorsed by PAHO/WHO, and validated by STRIDEBP according to current scientific evidence [29].

Lower-Limb Muscle Strength—Quadriceps and Hamstrings (QS-D, QS-ND; HS-D, HS-ND): Isometric muscle strength of the quadricep and hamstrings was assessed using Activ5 (Activbody Inc., Bellevue, WA, USA), which has excellent test–retest reliability (ICC > 0.90) [30]. Its ergonomic design allows the Activ5 to be used in multiple positions targeting different body regions and muscle groups, while the associated smartphone application facilitates clinical assessment and the programming of individualized exercise interventions. For quadriceps assessment, participants were seated with their back supported against a wall, hips and knees flexed at 90°, and feet placed shoulder-width apart on the floor. The Activ5 device was positioned under the foot, and participants were instructed to exert maximal voluntary isometric force. For hamstrings assessment, the same position was maintained; however, the device was placed behind the heel, and participants were instructed to press backward against the sensor. Three trials were performed on both the dominant and non-dominant limbs, with standardized verbal encouragement provided during each attempt. The highest value obtained across trials was used to represent maximal voluntary isometric strength, following commonly used procedures in clinical dynamometry protocols [30].

Sleep Quality—Pittsburgh Sleep Quality Index (PSQI): The PSQI consists of 19 self-reported items grouped into seven components: subjective sleep quality, sleep latency, sleep duration, sleep efficiency, sleep disturbances, use of sleeping medication, and daytime dysfunction. Each component is scored from 0 to 3, generating a global score ranging from 0 to 21, where higher scores indicate poorer sleep quality. The instrument has demonstrated moderate reliability and validity (Cronbach’s α = 0.70–0.85) [31].

Physical Activity—Global Physical Activity Questionnaire (GPAQ): The GPAQ includes 16 items assessing PA across three domains: work, transportation, and recreation. Participants report intensity, frequency, and duration of activity. One item evaluates daily sedentary time. The questionnaire has shown moderate reliability and validity (Spearman ρ = 0.73) [32].

Perceived Stress (PS): The Perceived Stress Scale includes 14 self-reported items assessing stress experienced during the previous month, scored on a 0–4 Likert scale (0 = never, 1 = almost never, 2 = sometimes, 3 = often, 4 = very often). Items 1, 2, 3, 8, 11, 12, and 14 are scored directly (0–4), while items 4, 5, 6, 7, 9, 10, and 13 are reverse scored (4–0) (30) [33]. The total score ranges from 0 to 56, with higher scores indicating greater perceived stress. Scores between 20 and 25 represent moderate stress; values above this threshold indicate high stress. The instrument demonstrates moderate reliability and validity (Cronbach’s α = 0.74) [34].

### 2.4. Procedure

Data collection was performed by final-year Physiotherapy students from the Technical University of the North, under the direct supervision of clinical researchers with experience in cardiovascular and functional assessment. The evaluators received structured training lasting approximately three months, which included theoretical components (study design, objectives, eligibility criteria, and measurement protocols) and supervised practical training in blood pressure measurement, muscle strength assessment, and questionnaire administration.

To reduce interobserver variability, procedures were standardized through pilot sessions conducted prior to the start of the study, using predefined operational protocols for each measurement. Throughout the data collection process, continuous supervision was maintained by the research team to verify adherence to procedures and the quality of the recorded data. However, it is acknowledged that the participation of multiple evaluators could introduce measurement variability, which was considered during the interpretation of the results.

### 2.5. Intervention/Assessment Protocol

**Figure 1:**
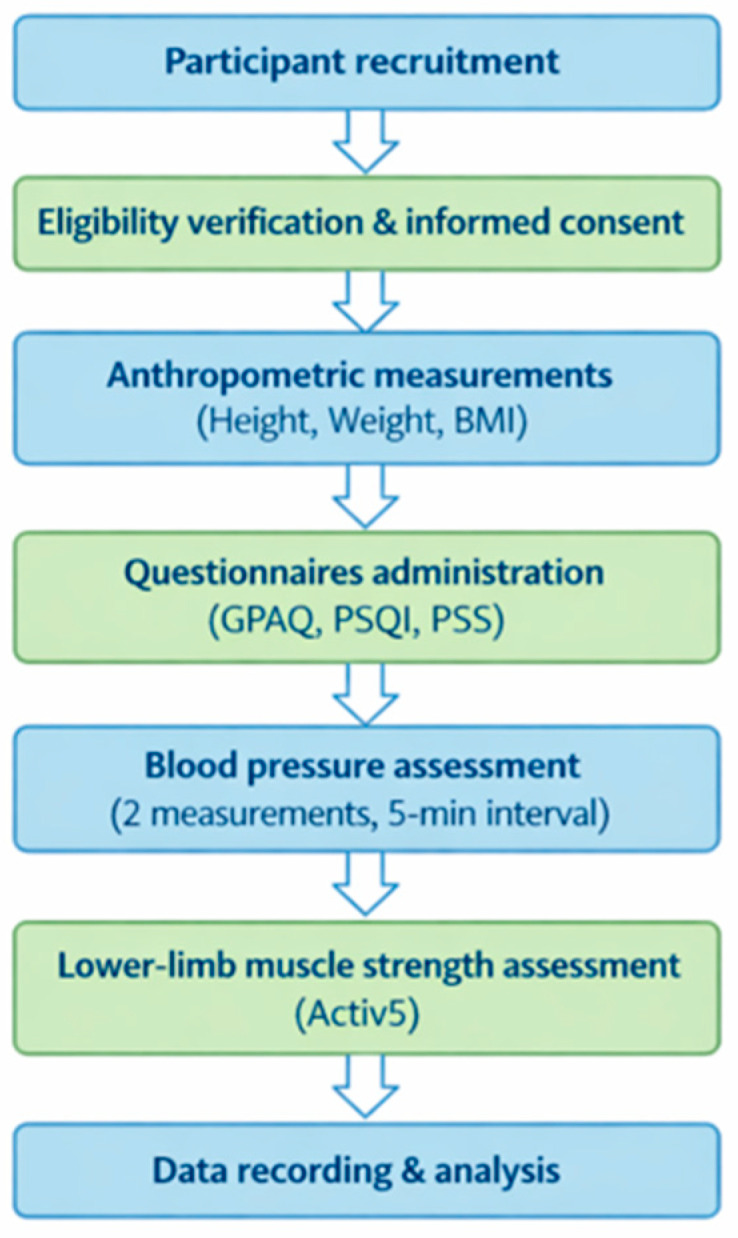
Overview of the assessment protocol followed during the single evaluation session.

The evaluation protocol lasted 40–45 min and consisted of four phases (Figure 1):

#### 2.5.1. Informed Consent

Eligibility criteria were verified, the purpose of the study was explained, and written informed consent was obtained from each participant. Additionally, a unique identification code was assigned to each patient to ensure data confidentiality.

#### 2.5.2. Data Collection and Questionnaires

General patient information was recorded, including age, sex, height, weight, and HTN diagnosis. The following questionnaires were administered: Global Physical Activity Questionnaire (GPAQ), Pittsburgh Sleep Quality Index (PSQI), and Perceived Stress Scale (PS).

#### 2.5.3. Blood Pressure Assessment

Participants remained seated with back support and rested for at least 5 min prior to measurement. Blood pressure was assessed using a validated automatic sphygmomanometer following standardized procedures. Two measurements were obtained, separated by a 5-min interval, and the mean systolic blood pressure (SBP) and diastolic blood pressure (DBP) values were recorded for statistical analysis.

#### 2.5.4. Muscle Strength Assessment

Muscle Lower-limb strength was evaluated using the Activ5 dynamometer. Quadricep strength was assessed first, starting with the dominant limb. Participants were seated on a firm surface with their back upright, hips and knees flexed at 90°, and feet fully supported on the floor. The device was positioned under the foot, and participants were instructed to exert maximal voluntary isometric force against the sensor. Standardized verbal encouragement was provided during each attempt.

For hamstring assessment, the same seated position was maintained; however, the device was placed behind the heel and stabilized against a fixed surface to prevent displacement. Participants were instructed to apply backward pressure with the heel while maintaining foot contact with the floor.

Three trials were performed for each limb, with a 30-s rest between attempts. The procedure was conducted on both dominant and non-dominant limbs, and the highest value obtained for each muscle group (QS-D, QS-ND; HS-D, HS-ND) was recorded for analysis to represent maximal voluntary isometric strength.

All assessments were conducted during a single session by trained evaluators following standardized procedures.

### 2.6. Data Analysis

A database was created and analyzed using jamovi statistical software (version 2.6.44). Quantitative variables were expressed as medians and interquartile ranges (Q1–Q3), whereas categorical variables were presented as frequencies and percentages. The normality of continuous variables was assessed using the Shapiro–Wilk test, demonstrating predominantly non-normal distributions. SBP was pre-specified as the primary out-come variable, whereas DBP and sex-stratified analyses were considered secondary out-comes.

Comparisons between men and women were performed using the Mann–Whitney U test, with the corresponding effect size reported. Associations between systolic blood pressure (SBP), diastolic blood pressure (DBP), and quantitative variables were evaluated using Spearman’s rho correlation coefficient, analyzed separately for men and women due to known physiological differences in cardiovascular regulation and muscle strength be-tween sexes. The magnitude of correlations was interpreted according to conventional criteria: trivial (<0.10), small (0.10–0.29), moderate (0.30–0.49), and large (≥0.50).

Subsequently, independent multivariable linear regression models were constructed for men and women, considering SBP and DBP as continuous dependent variables. Predictors included in the models (age, body mass index, lower-limb muscle strength variables, sleep quality, physical activity, and perceived stress) were selected a priori based on physiological plausibility, previous literature, and clinical relevance. Although several variables showed non-normal distributions, linear regression was considered appropriate due to the sample size and the evaluation of model assumptions through residual analysis, confirming linearity, homoscedasticity, independence, and an approximately normal distribution of residuals.

Overall model fit was evaluated using the F test, reporting the coefficient of determination (R^2^) and adjusted R^2^. Results were presented as unstandardized regression coefficients (β) with their corresponding standard errors (SE), 95% confidence intervals (95% CI), t values, and *p* values.

Multicollinearity among predictors was assessed using the variance inflation factor (VIF) and tolerance values, with VIF > 10 considered indicative of high collinearity. In the presence of moderate collinearity among muscle strength variables, individual coefficients were interpreted with caution. Missing data were minimal and handled using complete-case analysis. All analyses were performed using a statistical significance level of *p* < 0.05.

## 3. Results

The study population included 391 participants, comprising 165 men and 226 women. No statistically significant differences in age were observed between sexes (*p* = 0.146), with median ages of 60 years in men and 64 years in women. BMI was significantly higher in women compared with men (27.7 vs. 26.5 kg/m^2^; *p* = 0.010), with a small effect size. No significant differences were found between sexes in systolic blood pressure (SBP) or diastolic blood pressure (DBP), both showing trivial effect sizes. Significant differences were observed in lower-limb muscle strength, with higher values in men across all measurements. Quadricep strength demonstrated moderate effect sizes, whereas hamstring strength showed small effect sizes (Table 1).

**Table 1:** Characteristics of the Study Population.

Variables	Men (n = 165) Med (Q1–Q3)	Women (n = 226) Med (Q1–Q3)	U	*p*	Effect Size (r)
Age (years)	60 (49–73)	64 (48–78)	17,042	0.146	0.08
BMI (kg/m^2^)	26.5 (24.2–29.5)	27.7 (25.3–30.6)	15,809	0.010	0.15
SBP (mmHg)	143 (138–150)	140 (136–147)	16,656	0.071	0.10
DBP (mmHg)	89 (84–93)	88 (83–91)	17,104	0.162	0.08
QS-D (kg)	30 (22–44)	21.5 (16–32)	12,456	<0.001	0.33
QS-ND (kg)	30 (22–40)	21 (15–31)	12,794	<0.001	0.31
HS-D (kg)	13 (6–19)	9.5 (6–15)	16,174	0.025	0.13
HS-ND (kg)	10 (6–18)	7 (5–15)	15,734	0.008	0.15
GPAQ (MET-min/week)	1380 (650–3000)	840 (640–1740)	14,822	<0.001	0.20
PSQI (points)	6 (4–9)	7 (5–9)	15,890	0.010	0.14
PS (points)	27 (20–31)	25 (20–31)	17,910	0.505	0.03

n: number of participants (sample size); SBP: systolic blood pressure; DBP: diastolic blood pressure; QS-D: dominant quadricep strength; QS-ND: non-dominant quadricep strength; HS-D: dominant hamstring strength; HS-ND: non-dominant hamstring strength; GPAQ: Global Physical Activity Questionnaire; PSQI: Pittsburgh Sleep Quality Index; PS: perceived stress. Associations were analyzed using Spearman’s rank correlation coefficient (rho). The magnitude of correlations was interpreted according to Cohen’s criteria: trivial (<0.10), small (0.10–0.29), moderate (0.30–0.49), and large (≥0.50). Statistical significance was set at *p* < 0.05.

In men, systolic blood pressure (SBP) showed a moderate positive correlation with age (rho = 0.33; *p* < 0.001). Significant negative correlations were observed between SBP and lower-limb muscle strength variables, including dominant qquadricepstrength (QS-D; rho = −0.29; *p* < 0.001), non-dominant qquadricepstrength (QS-ND; rho = −0.22; *p* = 0.004), dominant hamstring strength (HS-D; rho = −0.15; *p* = 0.050), and non-dominant hamstring strength (HS-ND; rho = −0.17; *p* = 0.028), all with small effect sizes. No significant associations were found between SBP and BMI, physical activity (GPAQ), sleep quality (PSQI), or perceived stress (*p* > 0.05). For diastolic blood pressure (DBP), no statistically significant correlations were observed with the analyzed variables. A borderline association was identified with sleep quality (PSQI; rho = −0.15; *p* = 0.050), with a small effect size. Overall, the observed correlations were predominantly small, suggesting limited associations between the studied variables and blood pressure in men (Table 2).

**Table 2:** Associations of SBP and DBP with the study variables in men.

Variable	SBP (mmHg)				DBP (mmHg)	
	*p*	Rho	Strength	*p*	Rho	Strength
Age (years)	<0.001	0.33	moderate	0.735	0.02	trivial
BMI (kg/m^2^)	0.295	−0.08	trivial	0.453	0.05	trivial
QS-D (kg)	<0.001	−0.29	small	0.176	−0.11	small
QS-ND (kg)	0.004	−0.22	small	0.252	−0.12	small
HS-D (kg)	0.050	−0.15	small	0.112	−0.12	small
HS-ND (kg)	0.028	−0.17	small	0.797	−0.02	trivial
GPAQ (MET-min/week)	0.846	−0.02	trivial	0.050	0.07	trivial
PSQI (points)	0.258	−0.09	trivial	0.05	−0.15	Small
PS (points)	0.940	−0.01	trivial	0.09	0.260	trivial

SBP: systolic blood pressure; DBP: diastolic blood pressure; QS-D: dominant quadricepstrength; QS-ND: non-dominant quadriceps strength; HS-D: dominant hamstring strength; HS-ND: non-dominant hamstring strength; GPAQ: Global Physical Activity Questionnaire; PSQI: Pittsburgh Sleep Quality Index; PS: perceived stress. Associations were analyzed using Spearman’s rank correlation coefficient (rho). The magnitude of correlations was interpreted according to Cohen’s criteria: trivial (<0.10), small (0.10–0.29), moderate (0.30–0.49), and large (≥0.50). Statistical significance was set at *p* < 0.05.

In women, systolic blood pressure (SBP) showed significant negative correlations with dominant quadricep strength (QS-D; ρ = −0.16; *p* = 0.020), non-dominant qquadricepstrength (QS-ND; ρ = −0.14; *p* = 0.033), physical activity (GPAQ; ρ = −0.14; *p* = 0.034), and perceived stress (ρ = −0.20; *p* = 0.003). No other variables were significantly associated with SBP. Diastolic blood pressure (DBP) showed a small negative correlation with non-dominant hamstring strength (HS-ND; ρ = −0.19; *p* = 0.006). No other statistically significant associations were observed. Overall, the correlations identified in women were predominantly small, indicating limited relationships between the analyzed variables and blood pressure (Table 3).

**Table 3:** Associations of SBP and DBP with the study variables in women.

Variable	SBP (mmHg)			DBP (mmHg)		
	*p*	Rho	Strength	*p*	Rho	Strength
Age (years)	0.496	0.04	trivial	0.795	0.02	trivial
BMI (kg/m^2^)	0.12	−0.11	small	0.339	0.06	trivial
QS-D (kg)	0.020	−0.16	small	0.822	−0.02	trivial
QS-ND (kg)	0.033	−0.14	small	0.379	−0.06	trivial
HS-D (kg)	0.144	−0.09	trivial	0.036	−0.14	small
HS-ND (kg)	0.456	−0.05	trivial	0.006	−0.19	small
GPAQ (MET-min/week)	0.034	−0.14	small	0.055	−0.13	small
PSQI (points)	0.052	−0.13	small	0.828	−0.015	trivial
PS (points)	0.003	−0.20	small	0.910	0.009	trivial

SBP: systolic blood pressure; DBP: diastolic blood pressure; QS-D: dominant quadricep strength; QS-ND: non-dominant qquadricepstrength; HS-D: dominant hamstring strength; HS-ND: non-dominant hamstring strength; GPAQ: Global Physical Activity Questionnaire; PSQI: Pittsburgh Sleep Quality Index; PS: perceived stress. Rho: Spearman correlation coefficient. Correlation strength was interpreted according to Cohen’s criteria: trivial (<0.10), small (0.10–0.29), moderate (0.30–0.49), and large (≥0.50).

In men, the multivariable model for systolic blood pressure (SBP) showed a statistically significant overall fit (F(9, 155) = 3.82; *p* < 0.001), explaining 18% of the variability in SBP (R^2^ = 0.18; adjusted R^2^ = 0.134), indicating a modest explanatory capacity. Age demonstrated a significant positive association with SBP (β = 0.20; *p* < 0.001), while dominant quadricep strength (QS-D) showed a significant inverse association (β = −0.07; *p* = 0.017). No statistically significant associations were observed for BMI, non-dominant qquadricepstrength, hamstring strength variables, physical activity, sleep quality, or perceived stress (*p* > 0.05). Moderate to high multicollinearity was identified between hamstring strength variables (VIF ≈ 10), and therefore coefficients related to these predictors should be interpreted with caution (Table 4).

**Table 4:** Multivariable linear regression analysis for systolic blood pressure in men.

SBP (mmHg)	β	SE	t	*p*-Value	VIF
Age (years)	0.20	0.06	3.39	<0.001	1.27
BMI (kg/m^2^)	−0.12	0.21	−0.57	0.567	1.11
QS-D (kg)	−0.07	0.10	−2.40	0.017	4.14
QS-ND (kg)	0.27	0.11	0.69	0.488	4.67
HS-D (kg)	0.41	0.25	1.60	0.112	10.21
HS-ND (kg)	−0.31	0.25	−1.19	0.236	9.76
GPAQ (MET-min/week)	0.0006	0.0006	0.99	0.325	1.12
PSQI (points)	0.12	0.24	0.50	0.618	1.09
PS (points)	−0.13	0.10	−1.19	0.233	1.23
R = 0.43; R^2^ = 0.18; Adjusted R^2^ = 0.134; F(9, 155) = 3.82; *p* < 0.001					

SBP: systolic blood pressure; QS-D: dominant quadricep strength; QS-ND: non-dominant quadricep strength; HS-D: dominant hamstring strength; HS-ND: non-dominant hamstring strength; GPAQ: Global Physical Activity Questionnaire; PSQI: Pittsburgh Sleep Quality Index; PS: perceived stress. β: unstandardized regression coefficient; SE: standard error; VIF: variance inflation factor.

Diastolic blood pressure (DBP) in men showed a statistically significant overall model fit (F(9, 155) = 2.34; *p* = 0.017), explaining 12% of the variability (R^2^ = 0.12; adjusted R^2^ = 0.07), indicating a modest explanatory capacity. Dominant hamstring strength (HS-D) demonstrated a significant inverse association with DBP (β = −0.49; *p* = 0.002), whereas non-dominant hamstring strength (HS-ND) showed a significant positive association (β = 0.46; *p* = 0.004). Sleep quality (PSQI) was also inversely associated with DBP (β = −0.33; *p* = 0.032). No statistically significant associations were observed for age, BMI, qquadricepstrength variables, physical activity, or perceived stress (*p* > 0.05). However, moderate to high multicollinearity was identified between hamstring strength variables (VIF ≈ 10), and therefore coefficients related to these predictors should be interpreted with caution. Overall, the model suggests limited independent relationships between the analyzed variables and DBP in men (Table 5).

**Table 5:** Multivariable linear regression analysis for diastolic blood pressure in men.

DBP (mmHg)	β	SE	t	*p*-Value	VIF
Age (years)	0.005	0.035	0.15	0.879	1.27
BMI (kg/m^2^)	0.11	0.133	0.85	0.392	1.11
QS-D (kg)	−0.08	0.062	−1.40	0.163	4.14
QS-ND (kg)	0.08	0.070	1.15	0.250	4.67
HS-D (kg)	−0.49	0.159	−3.12	0.002	10.21
HS-ND (kg)	0.46	0.16	2.88	0.004	9.76
GPAQ (MET-min/week)	0.0005	0.0004	1.46	0.146	1.12
PSQI (points)	−0.33	0.15	−2.17	0.032	1.09
PS (points)	0.052	0.06	0.79	0.427	1.23
R = 0.35; R^2^ = 0.12; Adjusted R^2^ = 0.07; F(9, 155) = 2.34; *p* = 0.017.					

DBP: diastolic blood pressure; QS-D: dominant quadricepstrength; QS-ND: non-dominant qquadricepstrength; HS-D: dominant hamstring strength; HS-ND: non-dominant hamstring strength; GPAQ: physical activity; PSQI: sleep quality; PS: perceived stress; β: non-standardized regression coefficients; VIF: variance inflation factor.

In women, the multivariable model for systolic blood pressure showed a statistically significant overall fit (F(9, 216) = 4.48; *p* < 0.001), explaining 16% of the variability in SBP (R^2^ = 0.16; adjusted R^2^ = 0.13), indicating a modest explanatory capacity. Sleep quality (PSQI) demonstrated a significant inverse association with SBP (β = −0.61; *p* = 0.006), whereas perceived stress (PS) showed a significant positive association (β = 0.42; *p* < 0.001). No statistically significant associations were observed for age, BMI, quadricep and hamstring strength variables, or physical activity levels (*p* > 0.05). Severe multicollinearity was identified between HS-D and HS-ND (VIF > 10); therefore, coefficients related to hamstring strength should be interpreted with caution (Table 6).

**Table 6:** Multivariable linear regression analysis for systolic blood pressure in women.

SBP (mmHg)	β	SE	t	*p*-Value	VIF
Age (years)	0.03	0.03	0.82	0.411	1.28
BMI (kg/m^2^)	−0.25	0.15	−1.73	0.085	1.12
QS-D (kg)	−0.10	0.11	−1.01	0.313	6.62
QS-ND (kg)	−0.02	0.10	−0.27	0.783	6.13
HS-D (kg)	−0.08	0.21	−0.39	0.700	12.77
HS-ND (kg)	0.13	0.20	0.63	0.527	11.38
GPAQ (MET-min/week)	−0.00047	0.00073	−0.65	0.519	1.13
PSQI (points)	−0.61	0.22	−2.80	0.006	1.17
PS (points)	0.42	0.10	4.46	<0.01	1.10
Model fit: R = 0.40; R^2^ = 0.16; Adjusted R^2^ = 0.134; F(9, 216) = 4.48; *p* < 0.001.					

SBP: systolic blood pressure; QS-D: dominant quadriceps strength; QS-ND: non-dominant quadriceps strength; HS-D: dominant hamstring strength; HS-ND: non-dominant hamstring strength; GPAQ: Global Physical Activity Questionnaire; PSQI: Pittsburgh Sleep Quality Index; PS: perceived stress. β: unstandardized regression coefficient; SE: standard error; VIF: variance inflation factor.

Diastolic blood pressure (DBP) in women did not show a statistically significant overall model fit (F(9, 216) = 1.14; *p* = 0.338), explaining 5% of the variability (R^2^ = 0.05; adjusted R^2^ = 0.005), indicating minimal explanatory capacity. No statistically significant associations were observed between the studied predictors and DBP. Although non-dominant hamstring strength (HS-ND) reached statistical significance at the individual level (β = −0.28; *p* = 0.04), this finding should be interpreted with caution due to the lack of significance of the overall model and the presence of multicollinearity among muscle strength variables (VIF > 10). Overall, the model suggests limited associations between the analyzed variables and DBP in women (Table 7).

**Table 7:** Multivariable linear regression analysis for diastolic blood pressure in women.

DBP (mmHg)	β	SE	t	*p*-Value	VIF
Age (years)	−0.01	0.02	−0.41	0.679	1.28
BMI (kg/m^2^)	0.11	0.10	1.07	0.285	1.12
QS-D (kg)	0.10	0.07	1.38	0.167	6.62
QS-ND (kg)	−0.11	0.07	−1.63	0.104	6.13
HS-D (kg)	0.27	0.14	1.86	0.064	12.77
HS-ND (kg)	−0.28	0.14	−2.04	0.042	11.38
GPAQ (MET-min/week)	0.00006	0.00005	−1.20	0.230	1.13
PSQI (points)	−0.18	0.15	−1.19	0.235	1.17
PS (points)	0.03	0.06	0.44	0.656	1.10
R = 0.21; R^2^ = 0.05; Adjusted R^2^ = 0.005; F(9, 216) = 1.14; *p* 0.338					

DBP: diastolic blood pressure; QS-D: dominant quadricep strength; QS-ND: non-dominant qquadricepstrength; HS-D: dominant hamstring strength; HS-ND: non-dominant hamstring strength; GPAQ: Global Physical Activity Questionnaire; PSQI: Pittsburgh Sleep Quality Index; PS: perceived stress. β: unstandardized regression coefficient; SE: standard error; VIF: variance inflation factor.

## 4. Discussion

The present study analyzed the role of muscle strength, physical activity, perceived stress, and sleep quality in relation to systolic blood pressure (SBP) and diastolic blood pressure (DBP) in adults with hypertension. The main findings indicate that the associations between the studied variables and blood pressure differed according to sex. In men, SBP was independently associated with age and non-dominant quadricep strength, whereas DBP was primarily related to hamstring strength indicators and sleep quality. In women, SBP showed independent associations with perceived stress and sleep quality, while no significant multivariable model was identified for DBP. Overall, the explained variance of the models was modest, particularly for DBP, suggesting that functional and psychosocial factors may contribute differently to blood pressure regulation in individuals with hypertension and highlighting the multifactorial nature of this condition. The association between greater lower-limb strength and lower SBP highlights the potential relevance of muscular fitness as a functional marker of cardiovascular health. Conversely, the lower explanatory power observed for DBP may reflect the influence of other physiological, behavioral, or pharmacological factors that were not considered in the present model.

From a biopsychosocial model perspective within physiotherapy, these findings may contribute to the understanding of the potential interaction between muscular, behavioral, and psychosocial factors in individuals with hypertension. Although the cross-sectional design precludes causal inference, the observed associations suggest that muscle strength, perceived stress, and sleep quality could represent relevant components within a multidimensional assessment of patients with hypertension. These results should be interpreted within the exploratory nature of the study, considering the modest explanatory capacity of the models. Nevertheless, they support the rationale for further research examining the role of functional and behavioral factors in blood pressure regulation and the potential integration of therapeutic exercise-based approaches in comprehensive management strategies.

In our multivariable model, age showed a positive and significant association with systolic blood pressure (SBP) (β = 0.130; *p* < 0.001), which is consistent with evidence indicating that aging is related to structural and functional changes in the vascular system, such as increased arterial stiffness and reduced distensibility, factors that contribute to the progressive increase in SBP among adults with hypertension [35,36]. In contrast, BMI did not show a significant association with either SBP (β = −0.113; *p* = 0.342) or DBP (β = 0.126; *p* = 0.116), despite the fact that most participants were overweight. Although obesity and greater body adiposity have been linked to an increased risk of hypertension, in populations already diagnosed and possibly under antihypertensive treatment, the isolated effect of BMI may be attenuated, and variables such as fat distribution or metabolic profile may be more determinant than total body weight [37,38]. These findings reinforce the multifactorial nature of blood pressure control and highlight the need to interpret BMI within a broader clinical context.

In our study, greater lower-limb strength, particularly dominant quadricep strength, was associated with lower systolic blood pressure (SBP) values, suggesting that muscular characteristics in this region may play a functional role in hemodynamic regulation in individuals with hypertension. Previous research has reported associations between lower-limb functional performance and SBP, indicating that muscular capacity may reflect cardiovascular health status, although the magnitude of these relationships is generally modest. Additionally, higher SBP levels have been linked to accelerated decline in lower-limb function over time, supporting the existence of a bidirectional relationship between vascular health and muscular performance. From a physiological perspective, skeletal muscle function may influence vascular resistance, peripheral circulation, and metabolic regulation, and mechanisms that are particularly relevant in hypertensive and aging populations. Taken together, this evidence suggests that lower-limb strength may be considered a relevant functional marker in the context of hypertension, where its preservation could contribute to maintaining functional capacity and mitigating age-related physiological decline [39,40].

A possible explanation for these associations may be related to the integrated effects that muscle strength exerts on hemodynamics, vascular function, and autonomic regulation. Greater lower-limb muscle strength generally reflects better functional capacity, greater active muscle mass, and higher metabolic efficiency, factors that have been largely associated with improvements in endothelial function, increased nitric oxide availability, and reduced arterial stiffness. These adaptations favor more appropriate vasodilatory responses and more stable systolic blood pressure levels. Consistent with this rationale, a recent systematic review reported that both macrovascular and microvascular dysfunction are consistently associated with lower muscle mass, reduced strength, and poorer musculoskeletal function in adults, reinforcing the close relationship between vascular health and muscular health [41].

Complementarily, evidence from specific muscular interventions suggests that improvements in muscle function may contribute to vascular adaptations. For instance, inspiratory muscle training has been shown to reduce systolic blood pressure and enhance flow-mediated dilation, potentially through increased nitric oxide availability and reduced oxidative stress [42]. Likewise, in individuals with prehypertension and hypertension, different modalities of aerobic, resistance, and combined exercise have demonstrated comparable improvements in endothelial function and reductions in blood pressure, reinforcing the importance of active skeletal muscle as a modulator of vascular health [43]. In this context, physical exercise has been proposed as a fundamental tool in both the primary and secondary prevention of arterial hypertension due to its contribution to endothelial function and overall hemodynamic regulation [44].

Taken together, these findings suggest that muscle strength does not represent merely an isolated physical capacity but rather forms part of a physiological network integrating vascular control, autonomic regulation, and functional performance. This integrative framework may help explain the closer relationship observed between muscle strength and systolic components of blood pressure in the present study.

In the present research, perceived stress was independently associated with higher systolic blood pressure levels, particularly among women, which is consistent with previous evidence reporting moderate associations between stress and systolic blood pressure in some populations (r ≈ 0.47), compared with weaker relationships observed with diastolic blood pressure (r ≈ 0.20) [45]. Similarly, studies conducted in young adults with a family history of hypertension have reported moderate positive correlations between perceived stress and blood pressure control (r = 0.400; *p* < 0.001), as well as a significant contribution of stress in regression models (β = 0.400; *p* < 0.001) [46].

From a pathophysiological perspective, these findings are consistent with evidence indicating that chronic psychosocial stress may promote sympathetic nervous system activation and hypothalamic–pituitary–adrenal axis dysregulation, leading to hormonal and vascular alterations such as reduced nitric oxide bioavailability, endothelial dysfunction, and increased arterial stiffness. These mechanisms may increase hemodynamic load and provide a plausible biological explanation for the associations observed in the present study [47]. Taken together, this evidence suggests that perceived stress constitutes a relevant psychosocial determinant that may contribute to the maintenance of elevated blood pressure levels, particularly its systolic component, reinforcing the importance of considering psychological factors within the multidimensional management of hypertension.

We observed that poorer sleep quality was associated with higher diastolic blood pressure values, with small but statistically significant correlations, and remained an independent predictor within the multivariable model in men. These findings suggest that sleep disturbances may contribute to alterations in hemodynamic control in individuals with hypertension. This association has also been documented in clinical populations, where approximately 35.5% of individuals with hypertension present poor sleep quality, and those with elevated diastolic blood pressure show nearly a fourfold greater likelihood of sleep disturbances compared with individuals with lower blood pressure levels [48].

Complementarily, studies in older adults have reported that poor sleep quality is associated with an increased risk of hypertension and with adverse cardiovascular profiles across different sleep domains, including subjective quality, latency, and disturbances [49]. Additional evidence indicates that individuals with poor sleep quality may have a substantially higher likelihood of hypertension, with sleep quality emerging as a significant predictor in regression analyses [50]. From a physiological perspective, this relationship may be explained by increased nocturnal sympathetic activation, impaired autonomic regulation, oxidative stress, and endothelial dysfunction, mechanisms that contribute to elevated vascular resistance and attenuation of the normal nocturnal blood pressure decline. Taken together, these findings support the role of sleep quality as a clinically relevant component of the cardiovascular risk profile in hypertensive patients.

In our study, physical activity levels assessed using the GPAQ were not significantly associated with either systolic or diastolic blood pressure, and physical activity did not emerge as an independent predictor in the multivariable models. These findings suggest that self-reported habitual activity may not adequately reflect the physiological mechanisms involved in blood pressure regulation in individuals with hypertension, particularly in populations receiving medical treatment. However, substantial evidence indicates that when physical activity is structured and prescribed as a therapeutic intervention, its impact on blood pressure can be clinically meaningful. A recent meta-analysis reported that leisure-time physical activity is associated with clinically relevant reductions in both systolic and diastolic blood pressure in individuals with hypertension [12]. Complementarily, a systematic review and meta-analysis conducted in older adults with prehypertension and hypertension demonstrated that resistance training can reduce systolic blood pressure by approximately 6–7 mmHg and diastolic blood pressure by around 3 mmHg, suggesting consistent hemodynamic benefits when exercise programs are adequately structured [51]. Likewise, combined interventions integrating both aerobic and resistance training have also shown favorable effects on blood pressure levels in adults with hypertension [52]. Finally, recent reviews emphasize that exercise intensity, modality, program duration, and professional supervision are critical determinants of the antihypertensive effect, whereas self-reported measures of general physical activity may not adequately capture this impact [13]. Taken together, these findings suggest that the lack of association observed in our study may be more closely related to the self-reported nature of the GPAQ, potential treatment effects, and the cross-sectional design, rather than to the absence of a true physiological effect of exercise on blood pressure.

It is important to consider that the effects of physical activity on blood pressure are not uniform and largely depend on specific training variables, including exercise modality, intensity, volume, program duration, and adherence. Additionally, individual characteristics such as age, baseline functional capacity, degree of blood pressure control, presence of cardiometabolic comorbidities, and antihypertensive medication use may substantially modulate the hemodynamic response to exercise. These factors may partly explain the absence of significant associations observed in the present study when physical activity was assessed using a self-reported questionnaire. In this context, current evidence supports the need for personalized and periodized exercise prescriptions based on comprehensive clinical and functional assessment, rather than relying solely on general physical activity recommendations. Such an individualized approach may optimize both clinical effectiveness and safety in individuals with hypertension and aligns with contemporary physiotherapy models emphasizing patient-centered management and multimodal intervention strategies.

Taken together, our findings suggest that lower-limb muscle strength, perceived stress, and sleep quality may be differentially associated with systolic and diastolic blood pressure in individuals with hypertension, whereas self-reported physical activity did not show a significant association in this population. However, these results should be interpreted with caution due to the cross-sectional design, the use of self-reported instruments, the potential influence of antihypertensive medication, and the presence of collinearity among some predictors. Moreover, the proportion of explained variance was modest, particularly for diastolic blood pressure, reflecting the complex and multifactorial nature of blood pressure regulation.

Despite these considerations, the present findings have relevant clinical implications, as they reinforce the importance of assessing individuals with hypertension from a functional and biopsychosocial perspective, incorporating muscle strength evaluation, sleep quality, and stress management as complementary components within physiotherapeutic management. Future research should explore these relationships through longitudinal designs and structured intervention studies to better determine causal pathways and to identify how these variables may be optimized within therapeutic exercise programs and cardiovascular rehabilitation strategies.

## 5. Conclusions

Systolic blood pressure showed sex-specific association patterns in adults with hypertension. In men, SBP was primarily associated with age and quadricep muscle strength, whereas in women psychosocial factors, particularly sleep quality and perceived stress, demonstrated stronger independent associations after multivariable adjustment.

The associations between lower-limb muscle strength and blood pressure were generally small and attenuated after adjustment for potential confounders, suggesting that muscular functional capacity may contribute as a complementary, rather than determinant, factor in blood pressure regulation.

Although some predictors reached statistical significance, the explanatory models demonstrated modest predictive capacity (R^2^ ≤ 0.16), particularly for diastolic blood pressure, and moderate-to-high multicollinearity was observed among certain strength variables. These statistical limitations reduce the interpretability of individual regression coefficients and warrant cautious interpretation of the findings.

Overall, the results suggest that blood pressure variability in adults with hypertension is influenced by a combination of physiological and psychosocial factors; however, these variables should be considered complementary contributors rather than primary determinants. Longitudinal studies integrating cardiovascular, behavioral, and psychosocial domains are needed to clarify causal pathways and clinical relevance.

## Data Availability

The data presented in this study are available on reasonable request from the corresponding author. The data is not publicly available due to privacy and ethical restrictions.

## Abbreviations

HTN	Hypertension
BP	Blood Pressure
SBP	Systolic Blood Pressure
DBP	Diastolic Blood Pressure
BMI	Body Mass Index
QS-D	Dominant Quadricep Strength
QS-ND	Non-Dominant Quadricep Strength
HS-D	Dominant Hamstring Strength
HS-ND	Non-Dominant Hamstring Strength
PSQI	Pittsburgh Sleep Quality Index
GPAQ	Global Physical Activity Questionnaire
PS	Perceived Stress
CVD	cardiovascular disease
CVR	Cardiovascular Risk
WHO	World Health Organization
PAHO	Pan American Health Organization
PA	Physical Activity
